# Effects of periodic sensory perturbations during electrical stimulation on gait cycle period

**DOI:** 10.1371/journal.pone.0209781

**Published:** 2018-12-31

**Authors:** Kailey Nishimura, Eva Martinez, Alexander Loeza, Jessica Parker, Seung-Jae Kim

**Affiliations:** Department of Biomedical Engineering, California Baptist University, Riverside, California, United States of America; ISEP Instituto Superior de Engenharia do Porto, PORTUGAL

## Abstract

The spinal cord contains the neural circuitry needed to generate rhythmic walking motions, and afferent sensory feedbacks are involved in the control of locomotion. In this study, we examined the influence of periodic electrical stimulation on the change in gait cycle period during treadmill walking. 40 subjects walked on a treadmill while receiving periodic bursts of electrical stimulation at various perturbation periods (-20, -40, -60, +20, +40 milliseconds from their initial gait cycle periods). Eleven subjects received electrical stimulation to the hamstring, and 29 received electrical stimulation to the calf. Each subject completed four trials; two trials were conducted using high amplitude stimulation causing a slight degree of joint motion, and the other two trials were conducted using reduced amplitude stimulation which did not cause observable motion. Through the trials, we sought to answer the following questions: 1) does the amplitude of electrical stimulation have an effect on the level of entrainment? 2) does the stimulation site effect the level of entrainment? Entrainment refers to the synchronization of gait cycle period to the period of electrical stimulation. The results showed that entrainment was observed when the perturbation periods were induced relatively close to the subject’s initial gait cycle period. For both stimulation sites, entrainment was shown in 59% of subjects at +/- 20 milliseconds from the initial gait cycle period. With reduced amplitude, entrainment was still observed (51% all stimulation site groups at +/- 20 milliseconds). In addition, after-effects following electrical perturbation were present as seen by changes in the mean gait cycle period. Our results suggest that human locomotor control is organized with a semi-autonomous peripheral oscillator influenced by afferent information, and that electrical stimulation has the potential to be a simpler, and cost-effective tool for locomotion rehabilitation.

## Introduction

There is considerable evidence that the walking motion is controlled by a central pattern generator (CPG) [1,2], which is an anatomical nerve structure located in the spinal cord or lower brain involved in rhythmic stepping motions. Although rhythmic motion originates from the CPG, it can also be chaperoned by deep parts of the brain when necessary. However, is it important to note that the neurological control of rhythmic motion is significantly different from the neurological control of the movement of other parts of the body [3,4]. While the CPGs may operate in absence of afferent sensory feedbacks, it was suggested that the CPGs are inadequate for the generation of functional locomotor activity without afferent sensory inputs [5]. Afferent sensory inputs from knee and ankle joints play a crucial role in producing locomotor patterns [6], suggesting that the neural circuits responsible for periodic walking motion include networks necessary to integrate afferent sensory information into locomotor patterns generators.

One characteristic of locomotion is its ability to be entrained [7]. Entrainment is the process by which two systems become synchronized due to their interaction with each other. In regards to walking, entrainment is often associated with phase locking, which is the ability to establish a phase relationship with a perturbation. An external periodic afferent input can modulate rhythmic limb movements. Many studies have been conducted using perturbations to induce mechanical entrainment of cyclic walking motions for therapeutic applications [5,8–10]. The ability of an applied mechanical perturbation to entrain the locomotor rhythm has been observed mostly in cats, but the entrainment phenomenon is not restricted to the four-legged animal. It was also observed that a person’s gait cycle period can be influenced with the use of a mechanical robotic device that imposed ankle plantar-flexion torque [11] and vibratory stimulation [12]. The results from the prior studies on human locomotion demonstrate that humans have the ability to spontaneously synchronize a period of their gait cycle to that of an imposed perturbation.

The observation of entrainment due to external periodic perturbations may suggest that the control of human locomotion involves nonlinear and dynamic characteristics [13,14]. Spinal neural networks may play a role as nonlinear neuro-mechanical oscillator in coordinating walking patterns, and are also thought to be heavily influenced by afferent sensory feedback [15]. Thus, rhythmic limb movements due to vibratory or mechanically imposed perturbation may be attributed to the interaction with such a neuro-mechanical oscillator [16,17]. However, it remains unclear if providing sensory feedback would be sufficient enough to yield observable entrainment on treadmill walking. In previous studies on passive dynamic walkers, it seemed that minimal active ankle movement was required to yield coordinated walking on level ground [18]. In one study that utilized perturbations induced by mechanical robots, entrainment was observed possibly as a result from the periodic plantar-flexion motions that were aligned with the ankle pushing phase [11]. This raised the question of whether entrainment of locomotion could be driven by a biomechanical factor, not necessarily due to a nonlinear oscillator influenced by afferent sensory feedback.

Therefore, in this study, we investigated the effects of external periodic perturbation using electrical stimulation on gait entrainment while walking on a treadmill. In this study, “entrainment” refers to the synchronization of the gait cycle period to the period of electro-muscular perturbations. This study specifically aimed to answer the following questions: (1) does the amplitude of electrical stimulation have an effect on the level of entrainment, and (2) does the stimulation site (calf muscle vs. hamstring) effect the level of entrainment? An analysis was performed on the changes in the gait cycle period of healthy subjects by applying slight amounts of periodic electrical stimulation to the muscles involving in the ankle or the knee motion. In addition, we further investigated if electrical stimulation with minimal amplitude, enough to produce sensory feedback but not enough to produce ankle plantar-flexion movement or knee flexion-extension movement, would still induce a form of entrainment. Our hypothesis was that if a nonlinear oscillator such as a lower-level rhythmic central pattern generator contributed to the production of entrainment, it was expected that entrainment should still be observable even with lower amplitudes of electrical stimulation and regardless of the stimulating sites.

This specific technique of retraining muscles and nerve pathways through functional electrical stimulation (FES) training sessions should be highlighted because it has the potential to be used in gait rehabilitation for those who have difficulty walking, such as stroke patients and the elderly. We hope that waking attributes such as gait cycle period, stride length, and/or joint flexion range can be improved through these sessions, ultimately resulting in increased mobility, health, and independence for subjects. If successful, treadmill training in conjunction with FES could serve as a simpler and cost effective gait training system.

## Materials & methods

### Subject

40 healthy subjects (17 female; 23 male; aged 22.1 ± 2.6) with no neurological or physical disability participated in this study. Subjects were not considered for this study if they found the electrical stimulation uncomfortable while walking on a treadmill. This study was approved by the Institutional Review Board of California Baptist University. All subjects signed consent forms agreeing to willingly participate in experimental trials.

### General experiment setup

For all trials, each subject walked on a treadmill while hooked up to a commercially used electrical stimulator (MOTIONSTIM8, Krauth+Timermann, German), controllable through a computer program. Two 2” x 2” square electrodes were placed about 10 cm apart on one of two locations on the subject’s dominant leg: 1) the calf, or 2) the hamstring. The electrode pair location either actuated the knee by contracting the hamstring muscle or actuated the ankle by contracting the calf muscle. Stimulation locations for the hamstring group included the peroneal nerve, semitendinosus, semimembranosus, and biceps femoris. Stimulation locations for the calf group included the sural nerve, gastrocnemius, peroneus or soleus. Exact electrode location was dependent on the participant’s preference, but was generally restricted to axially aligned electrodes along the grain of the muscle.

Eleven participants were tested in the hamstring group, while 29 participants were tested in the calf group. A motion capturing device (Optotrak 3D Investigator, NDI, Canada) was used to measure each subject’s gait cycle period by reading the infrared marker placed on each subject’s right heel. The marker was seen by the capturing device in order for its positions and times to be located. The data retrieved in real time was then sent to a PC. A program developed with LabVIEW was used to retrieve the markers’ spatial position from the motion capturing system (Optotrak) through an API (application programming interface). Specifically, the LabVIEW program tracked the time of the heel strike by detecting the foot position and used this data to compute each subject’s gait cycle period. The LabVIEW program was also used to control the onset of electrical stimulation during the trials. The electrical stimulating device (MOTIONSTIM8) was a battery-powered electrical stimulation device. For added safety, a current-isolator was used in conjunction with the device.

### Procedure

Prior to the start of the trials, each subject was introduced to electrical stimulation to determine their preferred stimulation amplitude. The selected amplitude, used for the first two trials (the full amplitude condition), was intended to be close to the limit each subject could withstand while remaining comfortable walking. The electrical stimulus amplitude used for all subjects ranged between 30~50 mA for a duration of 0.1 seconds with frequency of 60Hz (200-μs biphasic pulses). An amplitude between 30~50 mA consistently produced muscle contractions (a slightly noticeable joint motion), however the stimulus amplitude did not produce a strong enough muscle force that could assist nor interfere with walking.

A five-minute introductory trial preceded experimentation. During this trial, the subject walked for 5 minutes total: the first 2 minutes with no stimulation and then for 3 minutes with electrical stimulation. This stimulation was synchronized to the subjects recorded gait cycle period with 0 ms offset. This introductory trial was introduced so that the subjects could become accustomed to walking on a treadmill with and without stimulation.

After the introductory trial, a total of four experimental trials were conducted over two days (two trails per each day). Each subject was instructed to walk on a treadmill at their selected walking speed while being hooked up to the MOTIONSTIM8 device via two electrodes. The first two experiments were conducted using full electrical stimulation amplitude and the last two experiments were conducted using reduced amplitude.

For the first experimental trial (10 minutes), subjects walked for four minutes without electrical stimulation. For the following 4.5 minutes, subjects walked with electrical stimulation at the predetermined amplitude. Gait cycle period was defined as the time from heel strike until the next heel strike on the right leg. The program recorded the subject’s average gait cycle period (τ_i_) over the last 30 steps taken during the initial four minutes of walking. The average gait cycle period was used to determine the baseline stimulation period. The electrical stimulation was then administered at perturbation periods (τ_d_) offset from the baseline gait cycle period by -20, -40, and -60 ms. Each perturbation period lasted 90-seconds. The electrical stimulus amplitude ranged between 30~50 mA for a duration of 0.1 seconds with frequency of 60Hz (biphasic pulses). For the last 90-seconds of the experiment subjects walked with no electrical stimulation. This was used to observe the after-effects of the induced perturbations.

After a ten-minute break, the second experimental trial was conducted (8.5 minutes) where the offset timing was positively increased. In this trial, following a four-minute initial period with no electrical stimulation, electrical stimulation was introduced at positive offsets of 20 and 40 ms respectively in 90-second intervals. These two experiments constituted one day of trials. Each subject came in on another day where the two trials were repeated with a reduced stimulation amplitude. The amplitude of the reduced stimulation was approximately half of the amplitude experienced in the first trials. The selected amplitude was intended to cause subjects to feel a tingling sensation, but not so much as to evoke noticeable ankle or knee motion.

### Data analysis

A gait cycle period (duration) was defined from the heel strike time data obtained from the LabVIEW program. In each trial, every right heel strike time was recorded and the gait cycle period (τ) was computed by subtracting the heel strike time of each gait from that of the previous gait.

To assess entrainment, a plot (phase plot) was created that shows the relative phase difference in time between the moment of the heel strike and the time when electrical stimulation was given. In the phase plot, each row indicates the occurrence of the heel strike during each perturbation cycle. Entrainment requires subjects’ gait cycle period to synchronize to the period of the perturbations. Thus, if entrainment occurred, the time of the heel strike in each gait was the same. If entrainment did not occur, the heel strike drifted relative to the stimulation period.

The average gait cycle periods were computed before (over only the last 30 strides), during, and after the electrical perturbation in each trial to see the effect of the electrical perturbation on change in gait cadence. The first five data points were excluded when computing the average value during and after the perturbation periods. The averages for each subject were then averaged (group mean) to examine the change from the initial gait cycle duration. This analysis was performed for both the increasing and decreasing perturbation periods. The average of the last 30 strides of the initial period was used as a base, represented as 100%, for which all other average gait cycle periods were compared to as a percentage (%) change. For the increasing and decreasing perturbation trials, separate one-way repeated-measure ANOVA was used to see if the last period without electrical stimulation showed a significant change in gait cycle period. A post-hoc paired comparison test with Bonferroni correction was used to compare the gait cycle period measured over the perturbation period where stimulation was applied to the initial gait cycle period (baseline) at a significance level of 5%.

## Results

**Fig 1:**
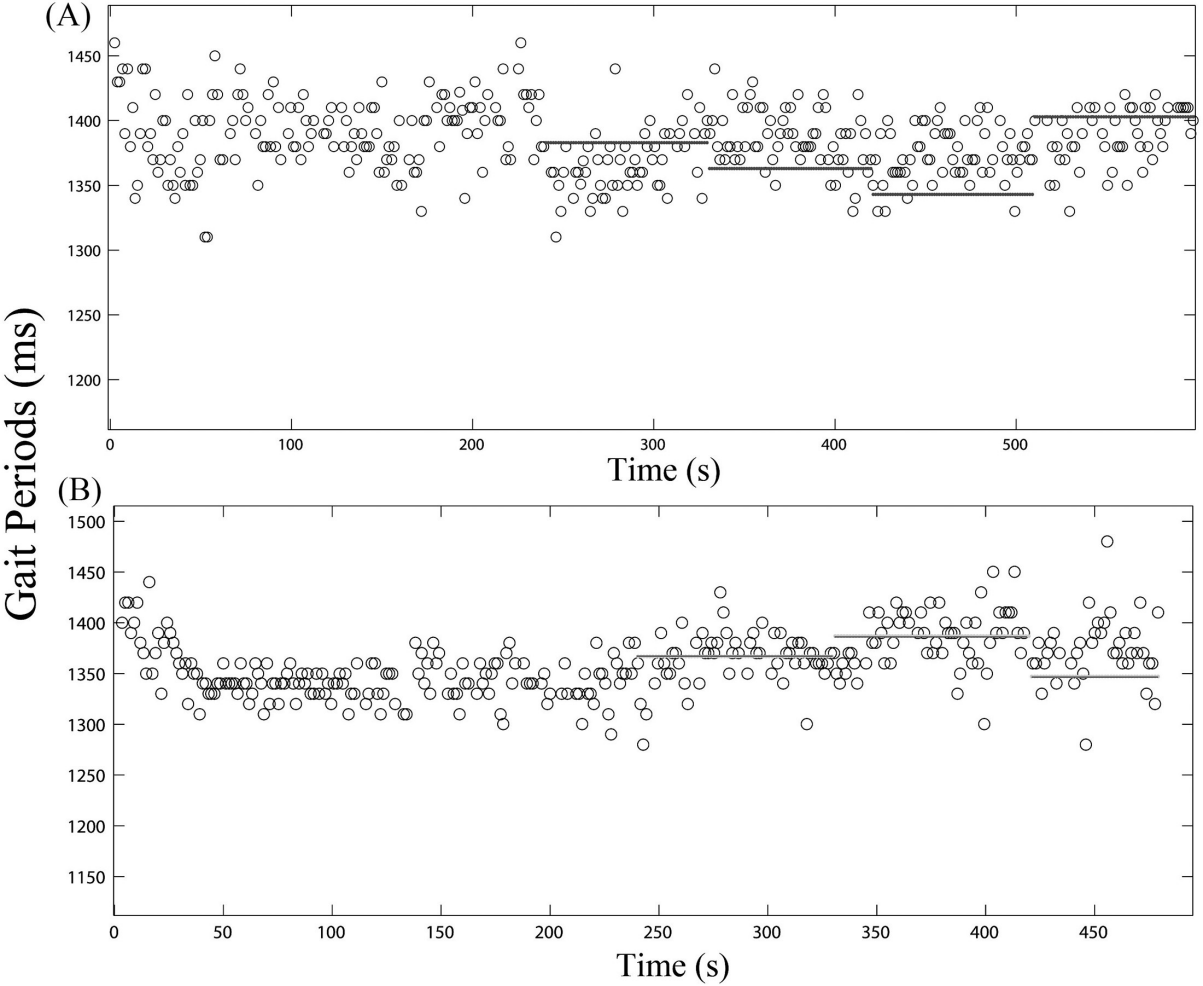
Typical plot of changes in gait cycle period measurements to perturbation. Example of changes in the gait cycle period for two different trials (negative (A) and positive (B) offset perturbations) obtained from a subject. The horizontal axis shows time; the vertical axis shows gait cycle period. Each circle represents the measured gait cycle period per gait. The trial period consisted of the first 4 minutes of walking with no electrical stimulation followed by 4.5 or 3 minutes of walking with periodic electrical stimulation and finished with the last 1.5 minutes of walking with no stimulation. (A) For the negative offset trial, 90 second perturbation periods of electrically stimulated pulses were sent at intervals of minus 20 ms, minus 40 ms, and minus 60 ms from the initial average gait cycle period, respectively. The black lines indicate the perturbation period at the time of the trial. The black line during the last 90 seconds indicates the initial average gait cycle period, which was obtained using the last 30 strides during the first four minutes of walking. (B) For the positive offset trial, 90 second perturbation periods of electrically stimulated pulses were sent at intervals of plus 20 ms and plus 40 ms from the initial average gait cycle period. The black line during the last 90 seconds indicates the initial average gait cycle period.

The overall trend in gait cycle period was first examined for each individual. Fig 1 shows the overall trend for the change in gait cycle period throughout the trial as a result of electrically stimulated perturbation of decreasing periods (-20, -40, and -60 of initial average gait cycle period) as shown in plot A and increasing (+20 and +40 of initial average gait cycle period) as shown in plot B for an individual subject. The horizontal black lines represent the perturbation period at that time of the trial.

**Fig 2:**
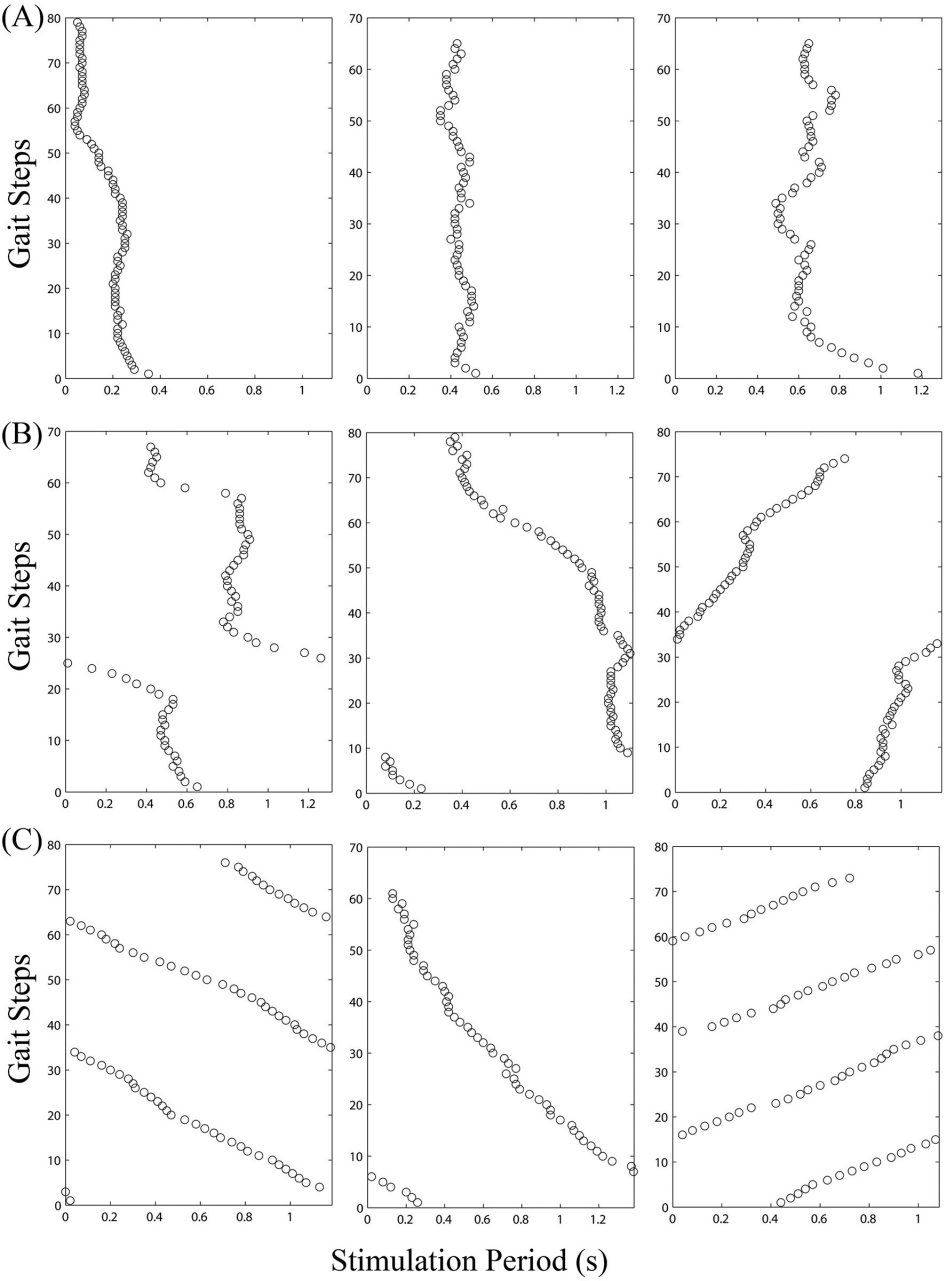
Typical phase plots for entrained gaits, some level of entrained gaits, and non-entrained gaits. Example of phase plots of subjects who exhibited entrainment (A), some level of entrainment (B), and non-entrainment (C) for a given condition. The circles represents the time of each heel strike relative to the stimulation period. A) Entrainment is represented by a vertical line of circles when gait cycles periods were stacked atop each other. B) Example of phase plots of a subjects who exhibited some level of entrainment for a given condition. C) Shows distinctive results of non-entrained to perturbation. When the heel strikes drift to the right, the actual gait cycle period was longer than the stimulation period. When the heel strikes drift to the left, the actual gait cycle period was shorter than the stimulation period.

In principle, entrainment requires a subject’s gait cycle period to converge to the period of the electrical perturbation. Convergence may occur with any phase relation between entrained gait and perturbation. Thus, if gait is entrained, synchrony will occur at a specific phase, which is called phase-locking. The phase plots, as shown in Fig 2A, shows examples of phase-locking obtained from different subjects. A phase plot shows the relative phase difference between gait and perturbation. The horizontal axis represents the stimulation period in seconds for a given offset condition, and the vertical axis represents the number of gaits that occurred during the perturbation period. Each circle represents the relative time in the gait cycle period when the subject had a heel strike with respect to the stimulation period. If entrainment occurred, the time of the heel strike in each gait was the same. A line of circles with no slope or drift indicates that the subject’s gait synchronized with the perturbations, hence being entrained (Fig 2A). If entrainment did not occur, the heel strike drifted relative to the stimulation period. Fig 2C shows phase plots of subjects who did not entrain to the perturbation. Additionally, if synchronization occurred at least one-third of the total electrical stimulation period, this was considered somewhat entrained, as shown in Fig 2B.

**Fig 3:**
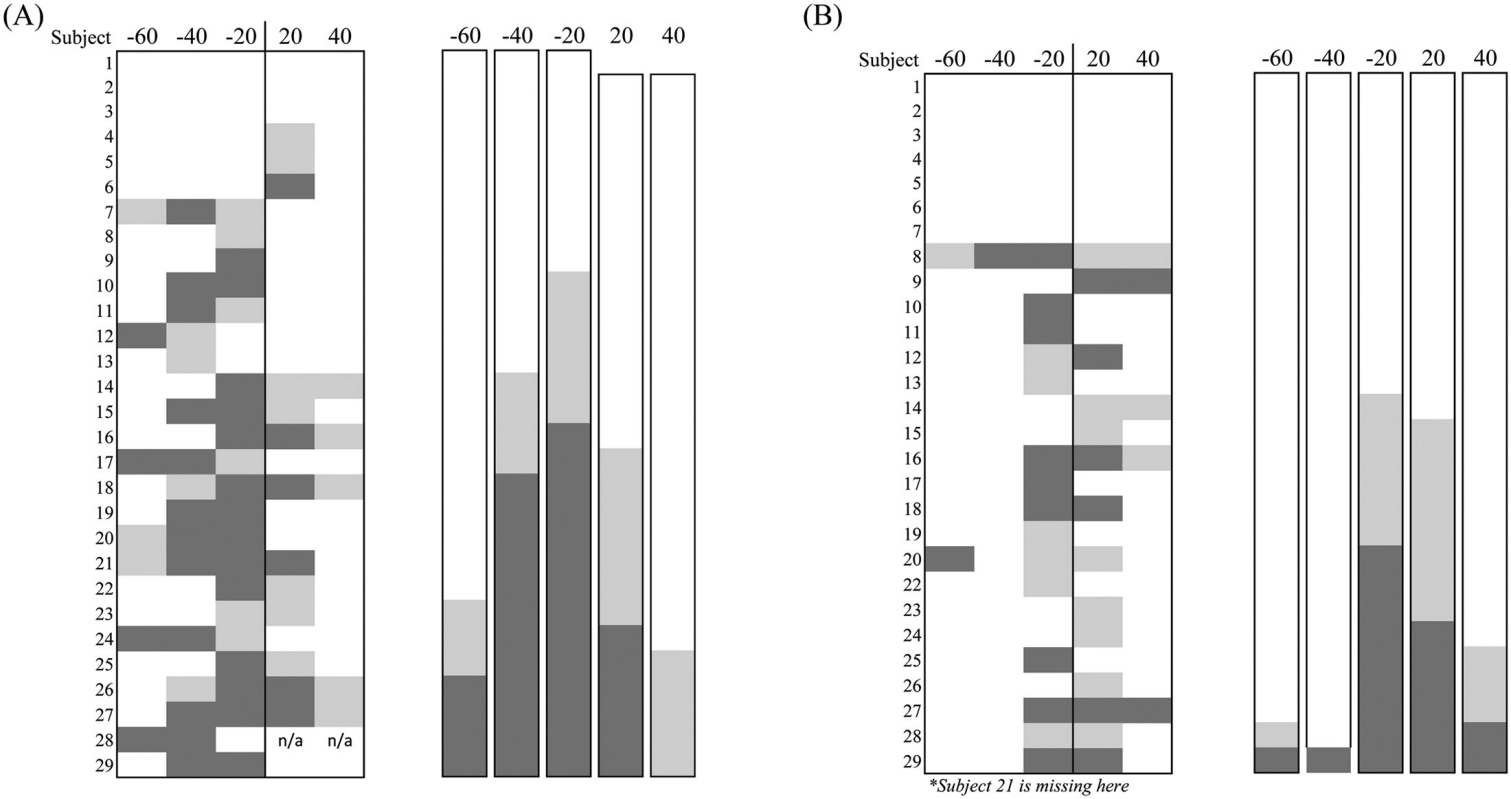
Effect of ankle perturbation period and stimulus amplitude on entrainment. Indicates the number of subjects who showed entrainment with the changes of ankle perturbation period. The darker and gray squares represent subjects that exhibited entrainment to the full and to some degree, respectively. The while squares represent subjects that were non-entrained. (A) Shows the effects of ankle perturbations at full amplitude. (B) Shows the effects of ankle perturbations at reduced amplitude. The plots on the left show the results for each individual subjects, whereas the plots on the right show the results without distinction from the subjects.

Fig 3 shows the results from the ankle perturbation condition for both full amplitude (A) and reduced amplitude stimulation (B). The plots on the left show the results for each individual subject, whereas the plots on the right show the results without distinction from the subjects. Both plots contain results from the two trials; decreasing and increasing electrical stimulation periods. The darker and gray squares indicated subjects that exhibited entrainment to the full and to some degree, respectively. The blank (white) squares represent non-entrainment. Fig 3A shows the results of 29 subjects under full amplitude. Entrainment, inclusive of some level of entrainment, was observed from 20 subjects (69%) on -20 ms offset condition, 16 (55%) on -40 ms, 7 (24%) on -60 ms, 13 out of 28 (47%) on +20 ms, and 5 (18%) on +40 ms. The results show that if the period of perturbation was sufficiently offset, entrainment was not observed. Reduced amplitude ankle perturbations, shown in Fig 3B, shows lower percentage of subjects whose gait was entrained. Entrainment, inclusive of some level of entrainment, was observed from 15 out of 28 subjects (54%) on -20 ms offset condition, 1 (4%) on -40 ms, 2 (7%) on -60 ms, 14 (50%) on +20 ms, and 5 (19%) on +40 ms.

**Fig 4:**
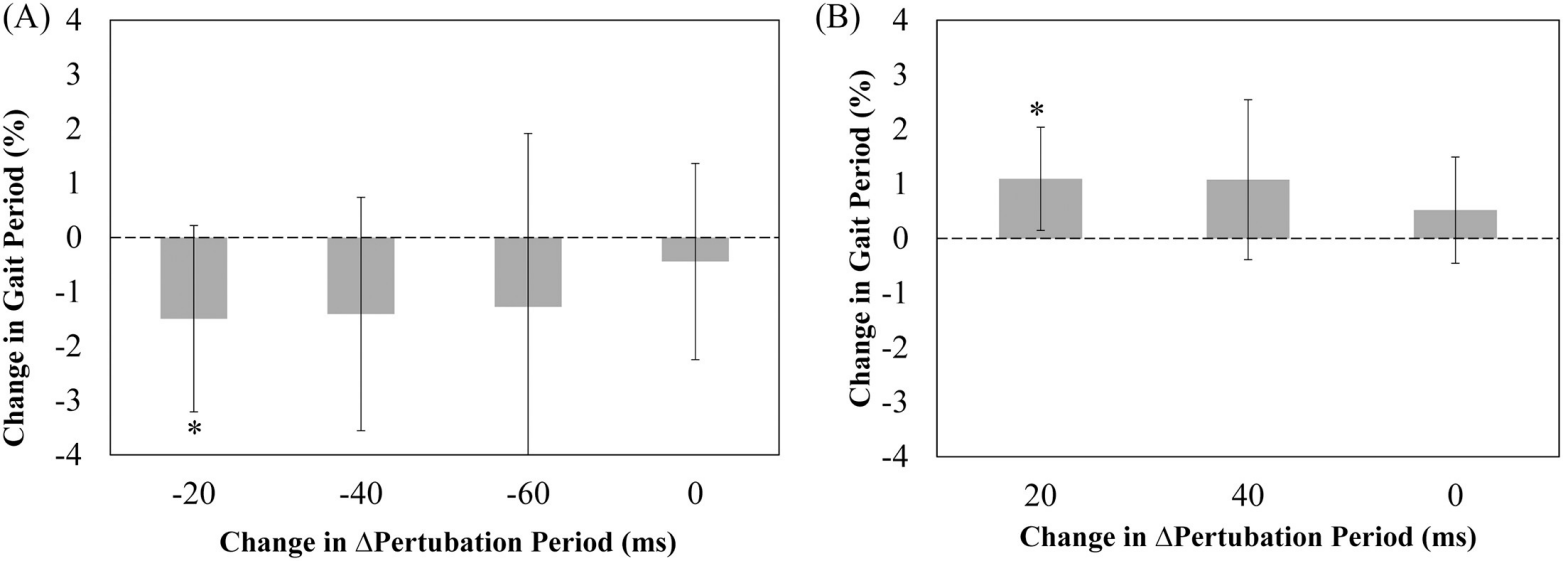
Group mean changes in gait cycle duration during ankle perturbation trial and aftereffect. (A) Shows the change in gait cycle period percentage of the full amplitude ankle perturbation condition with decreasing electrical stimulation period. The bars represent the average change in gait cycle period percentage for the 23 subjects who exhibited entrainment in at least one stimulation period of this condition (B) Shows the change in gait cycle period percentage of full amplitude ankle perturbation condition with increasing period electrical stimulation. The bars represent the average change in gait cycle period percentage for the 14 subjects who exhibited entrainment in at least one stimulation period of this condition. The asterisks (*) placed over the vertical bars shows the significant difference at p-value of p<0.05.

If entrainment occurred, the after-effects of perturbation were examined by comparing the mean gait cycle periods for each perturbation period and the last 90-second period where subjects walked without electrical stimulation. Not all subjects exhibited entrainment for any given stimulation period condition. Also, not many subjects responded under the conditions of reduced amplitude. Thus, Fig 4 shows the results for those subjects that showed entrainment for any stimulation period under the full amplitude condition. The average of the last 30 strides of the initial period was used as a base, represented as 100%, for which all other averages were compared to. The group means of gait cycle durations were then calculated for all subjects. Fig 4 also shows the results for the full amplitude condition of all 29 subjects for decreasing (plot A) and increasing (plot B) electrical stimulation periods. Each bar represents the group mean change in gait cycle duration for each perturbation period. The last bar is the average of the gait cycle durations for the finals period where subjects walked without electrical stimulation. This was used to analyze the after-effect from the induced perturbations imposed on the subject’s gait. The ANOVA revealed changes to gait cycle period during the increasing and decreasing perturbation periods, *F(4*,*88) = 3*.*774*, *F(3*,*39) = 4*.*015*, respectively.

**Fig 5:**
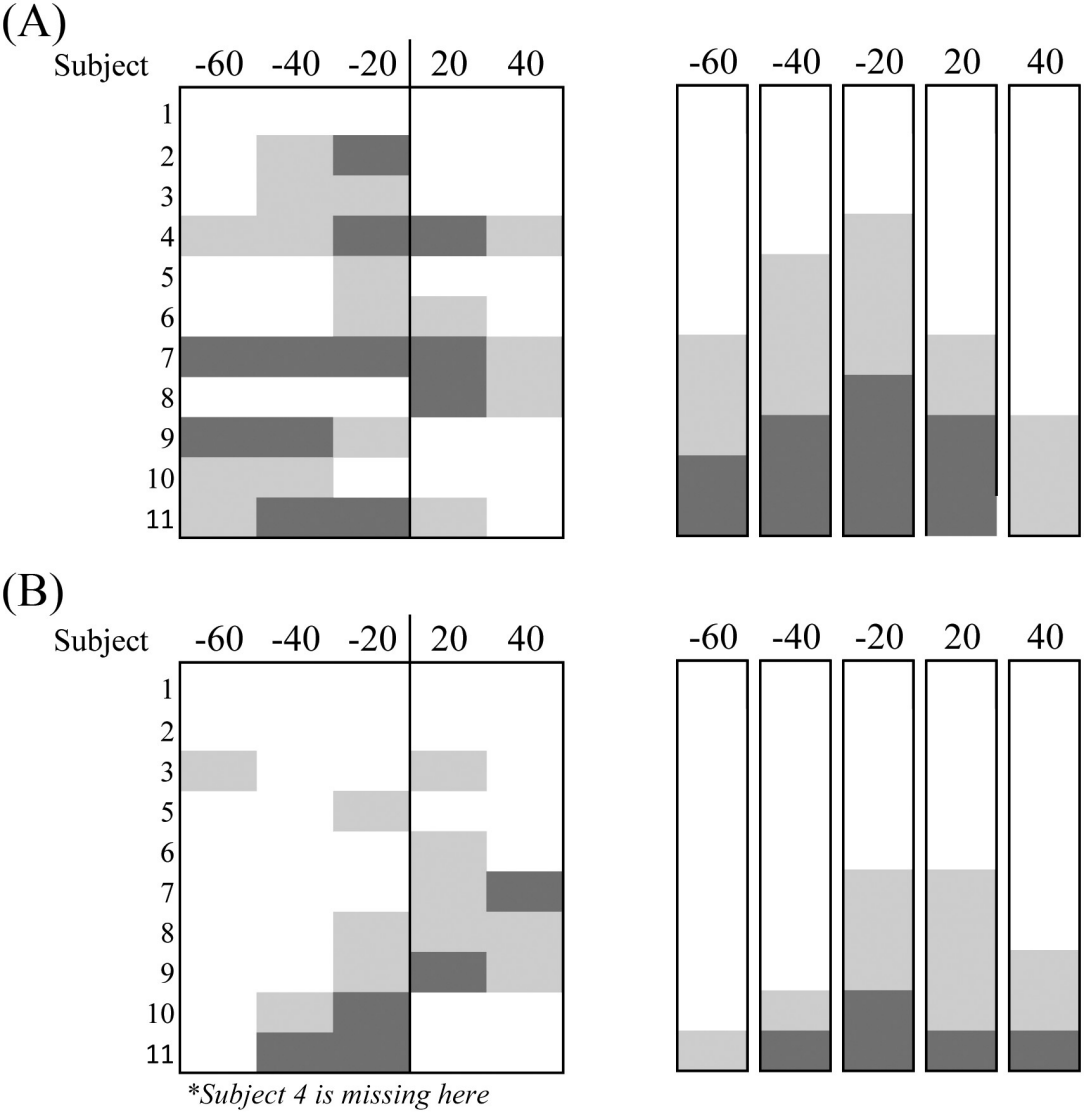
Effect of knee perturbation period and stimulus amplitude on entrainment. Indicates the number of subjects who showed entrainment with the changes of knee perturbation period. The darker and gray squares represent subjects that exhibited entrainment to the full and to some degree, respectively. The while squares represent subjects that were non-entrained. (A) Shows the effects of knee perturbations at full amplitude. (B) Shows the effects of knee perturbations at reduced amplitude. The plots on the left show the results for each individual subjects, whereas the plots on the right show the results without distinction from the subjects.

**Fig 6:**
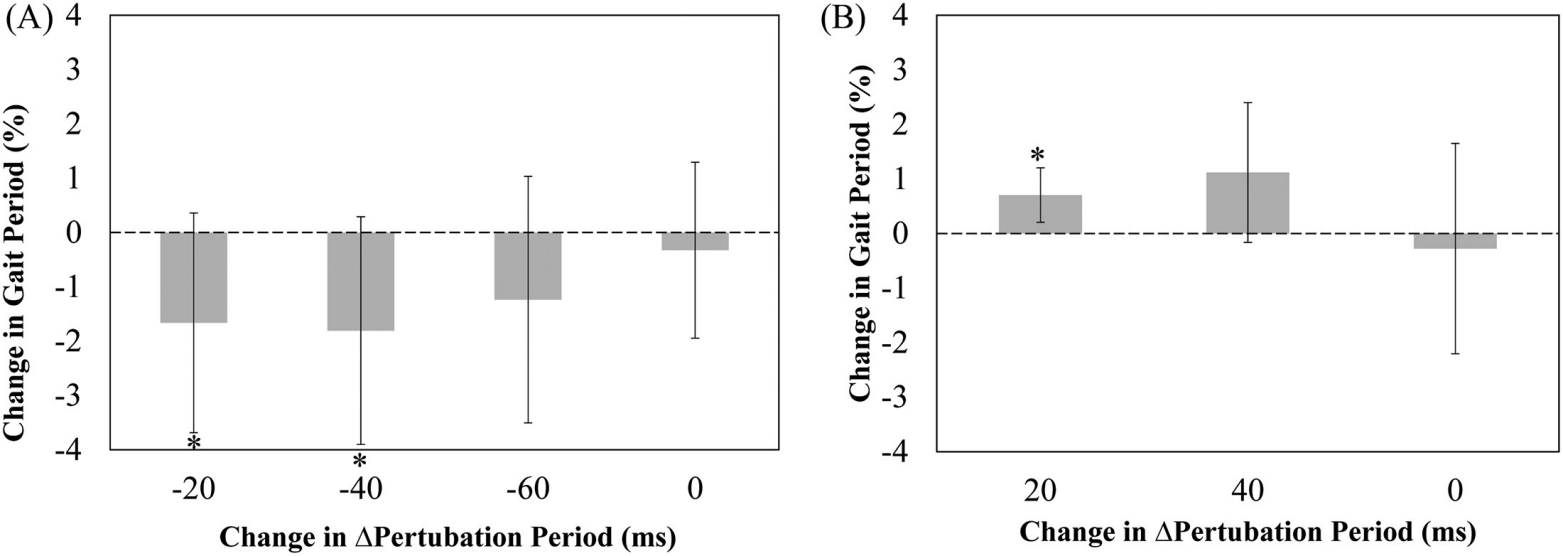
Group mean changes in gait cycle duration during knee perturbation trial and aftereffect. (A) Shows the change in gait cycle period percentage of the full amplitude knee perturbation condition with decreasing electrical stimulation period. The bars represent the average change in gait cycle period percentage for the 9 subjects who exhibited entrainment in at least one stimulation period of this condition (B) Shows the change in gait cycle period percentage of full amplitude knee perturbation condition with increasing period electrical stimulation. The bars represent the average change in gait cycle period percentage for the 5 subjects who exhibited entrainment in at least one stimulation period of this condition. The asterisks (*) placed over the vertical bars shows the significant difference at p-value of p<0.05.

Figs 5 and 6 show the results from the knee perturbation condition for both full and reduced amplitude conditions. Fig 5A shows the results of eleven subjects with full amplitude. Entrainment, inclusive of some level of entrainment, was observed from 8 subjects (73%) on -20 ms offset condition, 7 (67%) on -40 ms, 5 (45%) on -60 ms, 5 (45%) on +20 ms, and 3 (27%) on +40 ms. As the stimulation period increased or decreased away from the initial average, entrainment numbers dropped. Reduced amplitude ankle perturbations, shown in Fig 5B, showed a lower percentage of subjects whose gait was entrained. Entrainment, inclusive of some level of entrainment, was observed from 5 out of 10 subjects (50%) on -20 ms offset condition, 2 (20%) on -40 ms, 1 (10%) on -60 ms, 5 (50%) on +20 ms, and 3 (30%) on +40 ms. Fig 6 shows the change in gait cycle period percentage as a result of the full amplitude knee perturbation condition with decreasing and increasing electrical stimulation periods. The ANOVA revealed changes to gait cycle period only during the decreasing perturbation period, *F(4*,*32) = 3*.*087*.

## Discussion

In 1666, Christian Huygens, a Dutch physicist, discovered that when two clocks are mounted on the same surface their pendulum frequencies become synchronized [19]. It was further discovered that the transmitting medium which accounts for this synchronization is the vibration of the surface which the clocks lay on. This phenomenon was termed entrainment. The concept of the interaction between the periods of two systems was the basic idea behind which these experiments were conducted. The purpose of this study was to examine the influence of periodic electrical peripheral stimulation on gait entrainment during treadmill walking. In this study, entrainment refers to the synchronization of gait cycle period (duration) to the period of electrical stimulation. There is considerable evidence that rhythmic locomotor movements are generated by CPGs that can be activated by sensory feedback [20]. The rationale of using electrical stimulation was that somatosensory feedback from skin, muscles, and joints might have an important role in establishing some characteristics of walking rhythm movements. Thus, we investigated whether it was possible to entrain involuntary rhythmic movement patterns in healthy subjects with various stimulus amplitudes and stimulation sites. To assess whether rhythmic patterns could be entrained due to electrical perturbation, we measured the changes in the gait cycle period during trials, and aimed to answer the following questions: (1) does the amplitude of electrical stimulation have an effect on the level of entrainment, and (2) does stimulation site effect the level of entrainment?

All subjects walked on a treadmill at their chosen speed with applied electrical stimulation (0.1-s duration, 200-μs biphasic pulses at 60Hz) at periods different from their preferred gait cycle period (-20, -40, -60, +20, and +40 ms offset). The 40 subjects were divided into two groups (calf and hamstring groups) according to the site of stimulation. It was observed that significant entrainment occurred when the periodic perturbations were induced relatively close to the subject’s initials gait cycle period. Also, regardless of the site of stimulation, entrainment was still observed. Under the full amplitude condition, entrainment, including full and some level of entrainment, was shown in 58% of trials combined between both stimulating sites and at stimulation periods plus or minus 20 ms from the initial average gait cycle period (Figs 3A & 5A). As perturbation periods varied farther from the base line period, it was found that fewer subjects entrained to the periodic electrical perturbation. Entrainment typically requires gait cycle period to change to synchronize with the periodic perturbation. Because the treadmill speed was fixed, this required a compensatory change of step length. Typically, when entrainment occurs, a decrease in perturbation period would cause the subjects to walk faster. However, since the treadmill speed did not change, subjects had to alter their step length resulting in a smaller step length. In comparison, an increase in perturbation period typically would cause the subjects to walk slower, however due to the fixed treadmill speed, subjects had to increase their step length to entrain to the stimulation. Our results showed that subjects more easily entrained (70%) with a decrease in perturbation period (70% at both +/- 20 ms offset) than an increase in perturbation period (46% at both +/- 20 ms offset). This was probably because decreasing step length may be easier than increasing during treadmill walking.

It was also examined whether the site of stimulation affected the ability to be entrained. For both calf (ankle) and hamstring (knee) groups, entrainment was observed in 59% at a stimulation period plus and minus 20 ms. With reduced amplitude, entrainment was still observed (51% of cases for all stimulation site groups at plus and minus 20 ms offset), although there was a slightly lower percentage of subjects whose gait was entrained (Figs 3B and 5B). Regardless of the site of stimulation, entrainment was still observed. In our trials, full amplitude stimulation of the calf muscle induced slightly noticeable ankle plantar-flexion motions, and knee flexion motion for the hamstring groups, respectively. Although we did not measure muscle force/torque elicited by electrical stimulation, the stimulus amplitude did not produce a strong enough muscle force to assist nor interfere with walking. It was very unlikely that the low levels of muscle contractions affected joint kinematics. If entrainment in response to electrical perturbations were attributed to the electrically evoked motions that could assist the musculo-skeletal periphery to be entrained, the percentage of entrainment would be significantly different between the full amplitude and the reduced amplitude conditions. There would also be a difference between the calf and hamstring groups because it is reasonable to think that the movements in the ankle and knee joint are differently involved in the stepping pattern. Therefore, entrainment was likely due to the sensory sources.

Our findings are consistent with the results of prior studies conducted using mechanical robotic devices as a way to produce periodic ankle torque perturbation [11]. Previous studies demonstrated that human subjects entrained their gait cycle duration to the period of ankle torque pulses, only if the perturbation period was close to the subject’ preferred walking cadence (a narrow basin of entrainment), which was also observed in our study, especially where the treadmill speed conditions did not change.

The question of why entrainment occurs is complex. First, spinal neural networks have CPGs that strongly interact with afferent sensory feedback [6,8,12,21,22]. Therefore, periodic afferent signals evoked by peripheral stimulation may establish some features of entrainment. It was also suggested that the basal ganglia in the brain may have a significant role in entrainment. A strictly periodic perturbation supra-spinally provides the basal ganglia a time constant, which helps in creating a rhythmic anticipatory template [5,23]. However, several combinations of other factors, such as psychological and/or biomechanical factors, may also exhibit entrainment behavior. For instance, subjects were instructed to walk normally and not intervene with movements that might be induced by stimulation, but some subjects might unintentionally synchronize their gait cycle period to the perturbation period, even though they were not explicitly instructed to do so. Another biomechanical factor is associated with musculo-skeletal dynamics. Interaction between the gravitational mechanics of legs and their intermittent impact with the ground can produce coordinated passive walking [24], which possibly provides an environment for a cyclic behavior. Also, many fall and balance recovery strategies studies have suggested that perturbation in walking such as a slip, trip, or vibrotactile cuing, affects stepping strategies by altering stepping reaction times, foot clearance, and/or step velocity [25–27]. Thus, it might be possible that a person’s quick and voluntary accommodations to the electrically stimulated muscle contractions may have an effect on entrainment. Further studies are necessary in order to quantitatively address all possible effects.

Not all subjects exhibited entrainment for any given perturbation period condition. Among the subjects who showed entrainment for any given condition, none of the subjects showed a consistent response as the perturbation period decreased or increased (Figs 3A & 5A). A significant limitation of our experiments was that the treadmill had a fixed speed during trials. Thus, when entrainment occurred, subjects had to rely on a compensatory change of stride length. Consequently, anatomical consideration such as leg length might have been a hindrance to being more entrained to the perturbations. Related to this, another possible reason why not all subjects were sensitive to the perturbations is that our testing condition involved many of the mechanical constraint of treadmill walking. In other studies using electrical stimulation in a way to produce stepping motions [5,12], they tested during air-stepping condition or under anesthetized condition that were free from many of the mechanical constraints of normal walking. However, we tested entrainment under actual walking conditions in which the spinal neural networks had to handle various proprioceptive and sensorimotor afferents associated with limb loading and kinetics and kinematics of the limb. Consequently, the subjects might not be sensitive to the applied electrical stimulation in our testing condition. In addition, it was suggested that the contribution of sensory information to the CPGs are gait phase-dependent [28]. In our testing, the perturbation was not sent to a certain phase of walking, thereby not always guaranteeing the optimum response.

For rehabilitation purposes, the use of entrainment may be beneficial for patients to subconsciously increase their walking pace. The after-effect of the perturbations with the full amplitude condition was investigated to determine if subjects continued to walk with their adapted cadence after the completion of stimulation. As shown in Fig 4, the mean gait cycle period became shorter than the originally preferred value with decreasing electrical perturbation periods and longer than the originally preferred value with increasing electrical perturbation periods in ankle perturbation trials. For the knee perturbation trials (Fig 6), it appeared that there may have been some subtle differences in gait cycle period before and after perturbation, but we did not detect differences by a statistically significance level due to a large variability of entrainment. Although further study is needed to thoroughly investigate the after-effect of perturbation, it is worth stressing that prolonged training with electrical stimulation perturbation has the potential to lengthen the after-effect of the perturbation on the body. It is our hope that electrical stimulation can be used as an alternate cost-effective gait rehabilitation therapy, which can help patients to increase their gait cadence while training on a treadmill. In addition to the increase in walking speed, the use of electrical stimulation has also proven to increase muscle strength and stability [29]. Together the use of entrainment by electrical stimulation may be beneficial in assisting those with movement disabilities.

## Supporting information

S1 Data Set (ZIP)
